# Supplementary material for: Binding and Efficacy of Anti-Robo4 CAR-T Cells against Solid Tumors
Source: Biomedicines. 2022 May 30;10(6):1273. doi: 10.3390/biomedicines10061273 (PMC9220079; doi:10.3390/biomedicines10061273)
Supplement: Supplementary file 1 [file biomedicines-10-01273-s001.zip › biomedicines-1719789-supplementary.pdf]

Table S1 The amino acid sequence of anti-Robo4 scFv

CAR ID	Clone	Sequence				
CAR1	R-13	V_H	EVQLVESGGG	LVKPGGSLKL	SCAASGFTFS	SYAMSWVRQT
			PEKRLEWVAT	ISSGGSYTYY	PDSVKGRFTI	SRDNAKNTLY
			LQMSSLRSED	TAMYYCARND	YGYDFDYWGQ	GTTLTVSS
		V_L	DIVMTQSPAI	MSASPGEKVT	MTCSASSSVS	YMHWYQQKSG
			TSPKRWIYDT	SKLASGVPAR	FSGSGSGTSY	SLTISSMEAE
			DAATYYCQQW	SSNPPTFGGG	TKLELKR	
CAR2	R-14	V_H	EVQLVESGGG	LVKPGGSLKL	SCAASGFTFS	SYAMSWVRQT
			PEKRLEWVAT	ISSGGSYTYY	PDSVKGRFTI	SRDNAKNNLY
			LQMSSLKSED	TAMYYCARDS	HYRSRGYYFD	YWGQGTTLTV SS
		V_L	DIVMTQSPAI	MSASPGEKVT	ITCSASSSVS	YMHWFQQKSG
			TSPKLWIYST	SNLASGVPAR	FSGSGSGTSY	SLTISSMEAE
			DAATYYCQQW	SSNPPTFGGG	TKLELKR	
CAR3	R-18	V_H	QVQLQQSGAE	LVRPGTSVKI	SCKASGYTFT	NYWLGWVKQR
			PGHGLEWIGD	IYPGGGYTNY	NEKFKGKATL	TADTSSSTAY
			MQLSSLTSED	SAVYFCAREG	LRRGDYWGQG	TTVTVSS
		V_L	DIVMTQSPAI	MSASPGEKVT	MTCSASSSVS	YMHWYQQKSG
			TSPKRWIYDT	SKLASGVPAR	FSGSGSGTSY	SLTISSMEAE
			DAATYYCQQW	SSNPPTFGGG	TKLELKR	

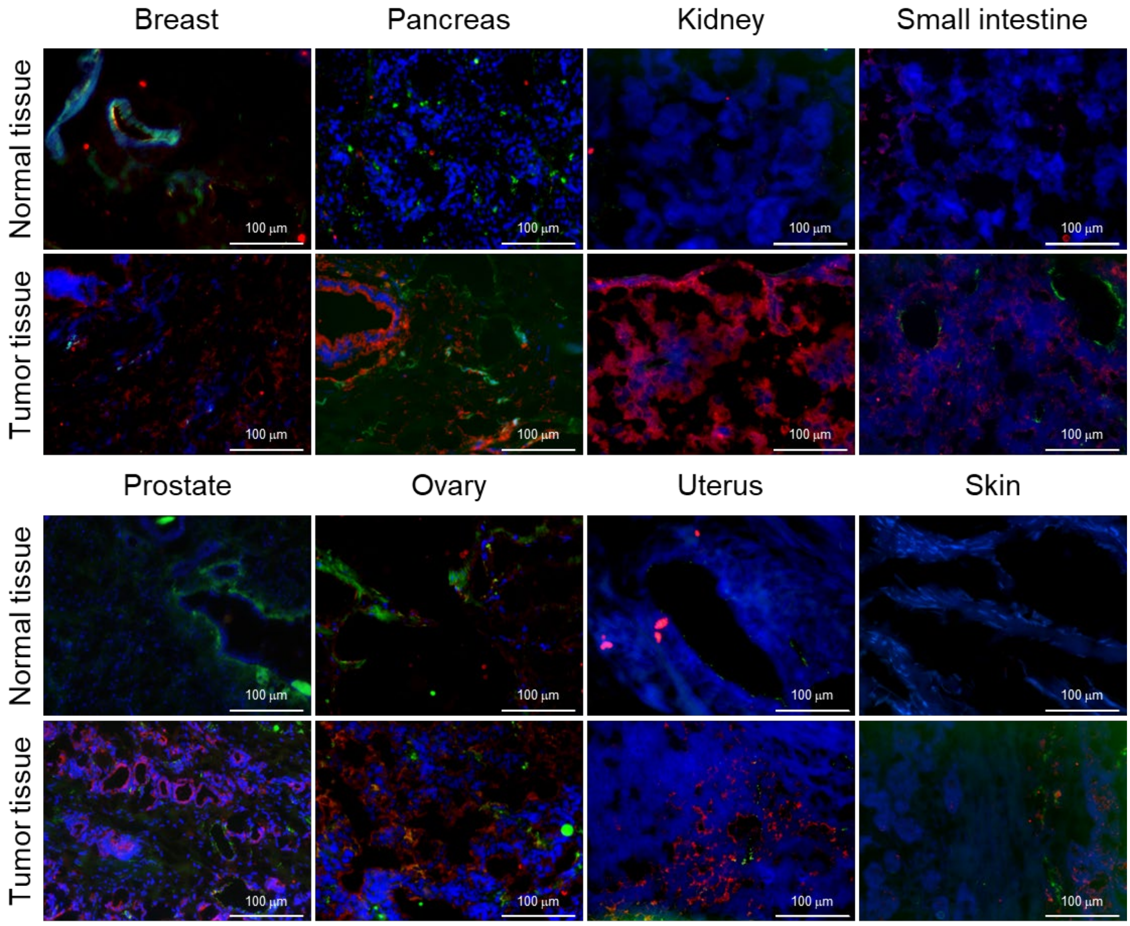

Figure S1. Expression of Robo4 in normal and tumor tissue using human tissue array. The rabbit anti-Robo4 antibody was synthesized by Syd Labs (Boston, MA, USA) based on the scFv of cloneR-14. The human Frozen Tumor and Normal Tissue Array (BioChain, Newark, CA, USA) was added with antibody dilution buffer containing anti-Robo4 antibody and mouse anti-human CD31 antibody (Clone WM59, BioLegend) and incubated at 4°C overnight. After removing the antibody solution, washing thoroughly with Tris-buffered saline containing 0.05% Tween-20, goat anti-mouse IgG highly cross-adsorbed secondary antibody, Alexa Fluor 488 (Thermo Fisher Scientific) and goat anti-rabbit IgG highly cross-adsorbed secondary antibody, Alexa Fluor 647 (Thermo Fisher Scientific) was added and incubated at room temperature for 2 h. After removing the antibody solution, plates were thoroughly washed with Tris-buffered saline containing 0.05% Tween-20 and mounted using ProLong Diamond Antifade Mountant with DAPI (Thermo Fisher Scientific). Fluorescence micrographs were acquired using a BZ-X800 microscope (Keyence). Representative images of immunofluorescence were shown with anti-Robo4 antibody (red), anti-CD31 antibody (green), and DAPI (blue).
